# Adipocyte Atrophy Mimicking Signet Ring Cell Carcinoma of the Gallbladder

**DOI:** 10.1155/2021/4676885

**Published:** 2021-10-31

**Authors:** Benjamin van Haeringen, Leo Francis, Emily Olive, Daniel James

**Affiliations:** ^1^Pathology Queensland Central Laboratory, Royal Brisbane and Women's Hospital, Herston, QLD, Australia; ^2^Faculty of Medicine, University of Queensland, Herston, QLD, Australia; ^3^Department of General Surgery, Royal Brisbane and Women's Hospital, Herston, QLD, Australia

## Abstract

Signet ring cell morphology may result from a variety of causes and ranges from a benign reactive phenomenon to being indicative of highly aggressive malignancy. Benign epithelial signet ring cell change is well described in a variety of tissues, but nonepithelial signet ring cell change is a rare morphologic adaptation of adipose tissue principally described in the setting of cachexia. The location of these atrophic adipocytes outside the plane of normal epithelial layers may raise concern for invasive or metastatic malignancy, and consideration of a benign reactive process is critical to avoid catastrophic overdiagnosis and overtreatment. Further, this change is itself associated with significant mortality related to the underlying cachexia and may be important to highlight to treating clinicians. Compared to malignant signet ring cell carcinoma, benign signet ring cell change is more likely to retain normal lobulated architecture without mass formation, lack significant atypia, have myxoid stroma with a prominent capillary network, and show positive staining S100 protein with negative staining for cytokeratins and mucin. To our knowledge, we present the first described case of nonepithelial signet ring cell change involving the gallbladder, detected as an incidental finding following routine cholecystectomy in an elderly cachectic man.

## 1. Introduction

Signet ring morphology is a histologic pattern commonly associated with clinically aggressive carcinoma and is a cause of concern to a practicing pathologist. Benign epithelial mimics are well described in a variety of tissues, often as a reactive phenomenon to injurious stimuli, and in the gallbladder manifest as characteristic univacuolated cells confined to the mucosa. Nonepithelial signet ring cell change is a rare morphologic adaptation of adipose tissue principally described in the setting of cachexia, which may simulate invasive malignancy and requires accurate recognition as a nonneoplastic process to avoid overdiagnosis and overtreatment.

## 2. Case Presentation

An elderly man presented to the hospital with severe epigastric pain, suspicious for acute cholecystitis. Subsequent magnetic resonance cholangiopancreatography was suggestive of acalculous cholecystitis, despite the patient not being critically unwell as usually seen with this entity. He was managed conservatively with antibiotics, resulting in the resolution of his symptoms. He was noted to be cachectic at admission, with a weight of 44 kg and body mass index (BMI) of 14.4 kg/m^2^. Despite gastroenterology and dietetics input, he had experienced progressive weight loss of approximately 20 kg over at least the previous 2.5 years, including 7 kg lost in the preceding 6 months. This was attributed to multiple dietary intolerances leading to a restricted diet and chronic diarrhoea. He also had a history of pseudomembranous colitis 20 years prior. He underwent an uncomplicated elective cholecystectomy, with intraoperative findings of a large, pendulous gallbladder and a global paucity of intra-abdominal adipose tissue.

**Figure 1:**
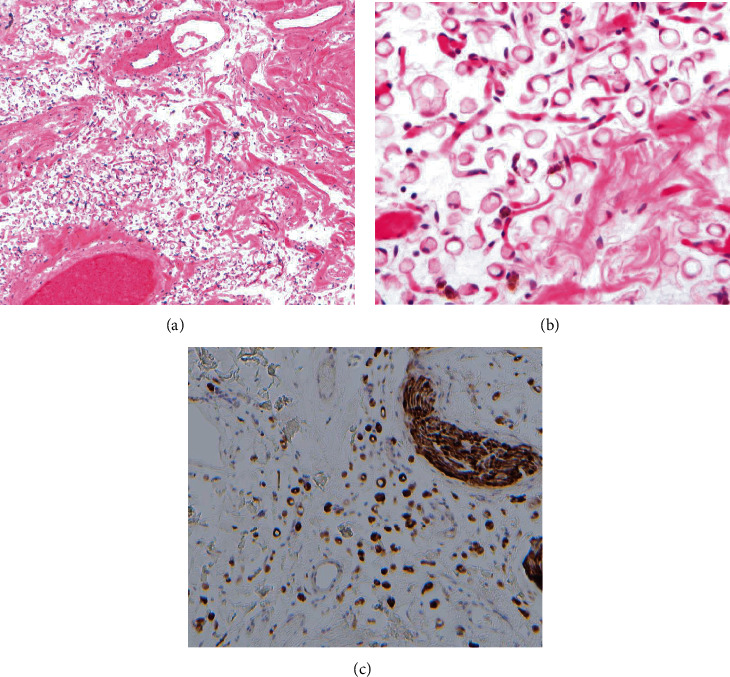
(a, b) The subserosal tissue exhibits a prominent population of signet ring-like cells, associated with oedema and a delicate capillary network. (c) The signet ring-like cells show strong nuclear and cytoplasmic positivity for S100 protein. A small nerve (upper right) is also reactive for S100 staining.

The gallbladder was macroscopically unremarkable, with wall thickness < 3 mm, no calculi, and no evidence of a polyp or mass. Histological assessment revealed extensive denudation of the surface epithelium with mild fibromuscular thickening of the wall, with residual columnar epithelium appearing within normal limits. Within the subserosa, there was a prominent population of cells with signet ring morphology, characterised by a large single intracytoplasmic vacuole displacing the nucleus to the cell periphery (Figures 1(a) and 1(b)). The cells lacked cytological atypia, had no connection to the mucosal surface, and did not show evidence of infiltrative growth. The background stroma was oedematous and contained a delicate vascular network of capillaries, with evidence of recent haemorrhage including scattered haemosiderin-laden macrophages.

The cells were negative for epithelial, endothelial, and melanocytic markers, including cytokeratin 7, cytokeratin 20, CDX2, SATB2, PAX8, D2-40, CD31, ERG, SOX10, and HMB-45, and negative for mucin on PAS-diastase stain. They showed strong nuclear and cytoplasmic staining for S100 protein (Figure 1(c)). They were therefore concluded to have derived from adipocytes, with morphological changes secondary to atrophy in the context of cachexia. There was no evidence of malignancy following submission of the entire gallbladder for assessment.

## 3. Discussion

Benign signet ring cell change is a well-recognised phenomenon, usually involving epithelial cells confined to the mucosa, which are suspected to arise from precursors with goblet cell differentiation [1]. In the gastrointestinal tract, they are typically associated with injurious stimuli such as ischaemia, traumatised polyps, or colitis (frequently pseudomembranous colitis) [1, 2]. Their appearance is often difficult to distinguish on cytological grounds from signet ring cell carcinoma, with obvious implications for prognosis and further treatment. Key features proposed to distinguish between the entities are that benign change is limited to the mucosa, lacks cytological or nuclear atypia, and is often associated with inflammatory or necrotic background changes [3, 4]. These cells are positive for pancytokeratin markers and mucin [3, 4]. In the current case, the history of pseudomembranous colitis is considered most likely incidental.

Nonepithelial signet ring cell change is a much rarer entity first described in 2006 [1], with only a handful of case reports since published, principally involving mesenteric fat and with no previous reports of gallbladder involvement. In most cases, the cells of concern were identified as atrophic adipocytes which uniformly stain positively for S100 protein and variably for calretinin, but negatively for pancytokeratin markers, histiocyte markers, vascular markers, melanocyte markers, other mesothelial markers, and mucin [1, 2, 5–8]. The cells lack cytologic or nuclear atypia and generally retain their normal lobulated, circumscribed architecture, although occasional pseudoinfiltrative growth has been seen and the cells may also be seen within lymph nodes and around nerves [6]. They are smaller than typical adipocytes, with mild size variation and rounded, thickened peripheral membranes, and are usually univacuolated although occasional multivacuolation has been described [5, 6]. The background stroma often shows conspicuous myxoid/mucoid change with a prominent delicate capillary network, and cells may show clustering around blood vessels [2, 5, 7]; this appearance has raised concern for myxoid liposarcoma [5]. Fibrosis may also be seen in association with the signet ring cells, which may accentuate lobulation [5, 6, 8].

In most cases, nonepithelial signet ring cell change has been associated with cachexia, with underlying causes including malignancy [5, 6, 8], mixed chronic inflammatory disease [7], and anorexia nervosa [5]. At admission, the BMI of these patients is reported to have ranged from 11.0 to 16.7 kg/m^2^. In two cases, background nutritional status was not reported, with the change being instead attributed to chronic localised ischaemia due to vascular insufficiency and fulminant *Clostridium difficile* colitis [1, 2]. It is possible that convergent morphologic alterations may be seen both in response to cachexia and severe inflammation. The finding is also associated with high mortality, with at least two of six reported antemortem cases culminating in death during hospital admission [2, 5], likely related to the underlying medical condition and/or cachexia which is itself associated with increased mortality [6].

In summary, this represents the first published case of nonepithelial signet ring cell change described in the gallbladder subserosa, in this instance due to adipocyte atrophy due to cachexia. This rare phenomenon has usually been described in patients with cachexia secondary to benign or malignant conditions and is associated with considerable mortality. Awareness of signet ring cell change (both epithelial and nonepithelial) is important to prevent inappropriate diagnosis of signet ring cell carcinoma. The most reliably reported histological features to distinguish this benign finding from malignancy are retained normal lobulated architecture without mass formation, lack of atypia, myxoid stroma with a prominent capillary network, and positive staining in signet ring-like cells for S100 protein with negative stains for cytokeratins and mucin.
